# PREOPERATIVE COMPUTED TOMOGRAPHY VOLUMETRY AND GRAFT WEIGHT ESTIMATION IN ADULT LIVING DONOR LIVER TRANSPLANTATION

**DOI:** 10.1590/0102-6720201700010011

**Published:** 2017

**Authors:** Rafael S. PINHEIRO, Ruy J. CRUZ-JR, Wellington ANDRAUS, Liliana DUCATTI, Rodrigo B. MARTINO, Lucas S. NACIF, Vinicius ROCHA-SANTOS, Rubens M ARANTES, Quirino LAI, Felicia S. IBUKI, Manoel S. ROCHA, Luiz A. C. D´ALBUQUERQUE

**Affiliations:** 1 Digestive Organ Transplantation Division, Department of Gastroenterology, Faculty of Medicine, University of São Paulo, São Paulo, SP, Brazil;; 2 Transplantation Division, Department of Surgery, University of L'Aquila; San Salvatore Hospital, Italy;; 3 Department of Radiology, Faculty of Medicine, University of São Paulo, São Pauloi, SP, Brazil.

**Keywords:** Liver transplantation, Living donors, Multidetector Computed Tomography.

## Abstract

**Background:**

Computed tomography volumetry (CTV) is a useful tool for predicting graft weights (GW) for living donor liver transplantation (LDLT). Few studies have examined the correlation between CTV and GW in normal liver parenchyma.

**Aim:**

To analyze the correlation between CTV and GW in an adult LDLT population and provide a systematic review of the existing mathematical models to calculate partial liver graft weight.

**Methods:**

Between January 2009 and January 2013, 28 consecutive donors undergoing right hepatectomy for LDLT were retrospectively reviewed. All grafts were perfused with HTK solution. Estimated graft volume was estimated by CTV and these values were compared to the actual graft weight, which was measured after liver harvesting and perfusion.

**Results:**

Median actual GW was 782.5 g, averaged 791.43±136 g and ranged from 520-1185 g. Median estimated graft volume was 927.5 ml, averaged 944.86±200.74 ml and ranged from 600-1477 ml. Linear regression of estimated graft volume and actual GW was significantly linear (GW=0.82 estimated graft volume, r^2^=0.98, slope=0.47, standard deviation of 0.024 and p<0.0001). Spearman Linear correlation was 0.65 with 95% CI of 0.45 - 0.99 (p<0.0001).

**Conclusion:**

The one-to-one rule did not applied in patients with normal liver parenchyma. A better estimation of graft weight could be reached by multiplying estimated graft volume by 0.82.

## RESUMO

**Racional:**

A volumetria por tomografia computadorizada (VTC) é uma ferramenta útil para a previsão do peso do enxerto (PE) para o transplante hepático com doador vivo (TFDV). Poucos estudos examinaram a correlação entre o VTC e PE no parênquima hepático normal.

**Objetivo:**

Analisar a correlação entre VTC e PE em uma população adulta de doadores para o TFDV e realização de revisão sistemática dos modelos matemáticos existentes para calcular o peso de enxertos hepáticos parciais.

**Métodos:**

Foram revisados retrospectivamente 28 doadores consecutivos submetidos à hepatectomia direita para o TFDV entre janeiro de 2009 a janeiro de 2013. Todos os doadores eram adultos saudáveis ​​com VTC pré-operatório. Os enxertos foram perfundidos com solução de preservação HTK. O volume estimado foi obtido por VTC e estes valores foram comparados com o peso real do enxerto, o qual foi aferido depois da hepatectomia e perfusão do enxerto.

**Resultados:**

A mediana do PE real foi de 782,5 g, média de 791,43±136 g, variando de 520-1185 g. A mediana do volume estimado do enxerto foi de 927,5 ml, média de 944,86±200,74 ml e variou de 600-1477 ml. A regressão linear volume estimado do enxerto e PE real foi significativamente linear (PE=0.82 do volume estimado do enxerto, r2=0,98, declive=0,47, desvio-padrão de 0,024 e p<0,0001). Correlação linear de Spearman foi de 0,65, com IC de 95% do 0,45-0,99 (p<0,0001).

**Conclusão:**

A regra de "um-para-um" não deve ser empregada em pacientes com parênquima hepático normal. A melhor estimativa do peso do enxerto hepático de doador vivo pode ser alcançado através da multiplicação do VTC por 0,82.

## INTRODUCTION

The first living donor liver transplantation (LDLT) was performed in 1989 at our Institution^15^. Since then, surgical techniques and patient´s care improvements have established LDLT as a valid option of overcoming the shortage of deceased donors. 

Initially, LDLT was exclusive to pediatric patients, although over time it also became good option to adult recipients as well. One of the major concerns of this procedure is to provide an adequate graft volume while preserving a suitable hepatic remnant for the donor. Reduced grafts are associated with small for size syndrome and poor outcomes. In this particular setting, preoperative radiological evaluation of liver volume is the standard method for donor evaluation, aiming minimization of unnecessary risks.

Therefore, accurate assessment of the volume of the liver and its lobes prior to surgery is mandatory. Computed tomography volumetry (CTV) is a useful tool for predicting graft weights (GW) for LDLT^16^ ^,^ ^18^. However, for the conversion of volumetric weight, the rule of "one-to-one" does not offer good results due that this rule was based in the weight using cirrhotic livers^20^. Fw studies have examined the correlation between CTV and GW in normal liver parenchyma. 

The aim of this paper was to analyze the correlation between CTV and GW in an adult LDLT population, and also provide a systematic review of the existing mathematical models to calculate partial liver graft weight or volume, based on different radiological parameters. 

## METHODS 

Between January 2009 and January 2013, 28 consecutive donors undergoing right hepatectomy for LDLT were retrospectively reviewed. All donors were healthy adults with preoperative CTV. 

Hepatectomy cutting plane was guided by ischemic line after a short period (about 2 min) of right pedicle clamping (i.e. right hepatic artery and right portal vein). Right hepatic graft, including middle hepatic vein, was used in all but two cases. Grafts were perfused via the right portal vein with cold histidine-tryptophan-ketoglutarate (HTK) solution as soon as hepatectomy was completed. The solution was drained from the liver and a pre-calibrated digital scale was used to define the actual GW.

### Preoperative measurement of the liver volume 

Multidetector computed tomography (CT) images were obtained with four different devices: CT Discovery 750 HD (GE Medical Systems, Milwaukee, Wisconsin, USA), CT Light Speed (GE Medical Systems, Milwaukee, Wisconsin, USA), CT IDT MX8000 (Philips Healthcare, Cleveland, Ohio, USA) and CT Brilhance 64 (Philips Healthcare, Cleveland, Ohio, USA), with a 1-mm slice thickness and 70 s after the intravenous administration of iodine contrast media (portal venous phase). These data were used for CT volumetry measurements. The scanning parameters were as follows: 120 kV and mAs appropriate to body habitus.

**FIGURE 1:**
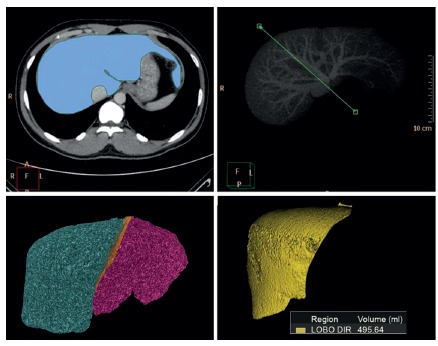
Images from pre operative CT hepatic graft volumetry of living donor: A) delimitation of whole hepatic area for CT volumetry; B) green line demarking medium hepatic vein, this line splits right and left hepatic lobes; C) right and left hepatic lobes; D) virtual right hepatectomy with estimated volume

The right lobe graft volume (GV) was measured by tracing a line on the right of the middle hepatic vein, thus defining the virtual hepatectomy plan. The perimeters of the liver and the graft were outlined by hand tracing on each slice by an abdominal radiologist. The enclosed area was calculated with image analysis software Volume Viewer (GE Medical Systems, Milwaukee, Wisconsin, USA). The liver volume (in milliliters) was then obtained as the sum of all areas from the intervals of the serial CT slices (Figure 1).

Estimated graft volume (EGV) was obtained by CTV, and these values were compared to the actual GW (AGW), which was defined after liver harvesting and perfusion. 

### Statistical analysis

Values are shown as mean±standard deviation. Statistical analysis was performed using R version 2.15.2 (The R Foudantion for Statistical Computing). A value of p<0.05 was considered significant. Relation between estimated GV and AGW was determined by use of linear regression analysis; furthermore, linear correlation of Spearman and its 95% confidence interval were presented.

## RESULTS

The donor's demographics and perioperative parameters are summarized in Table 1. Blood products transfusion was not necessary in any of the donor's surgeries. 

**TABLE 1:** Donor´s characteristics: continuous variables are reported as medians and ranges; categorical variables are reported as numbers and percentages.

Variables	Data
Age (years)	29 (17 - 43)
Male gender (%)	21 (75)
Max postoperative total bilirrubine (mg/dl)	2.74 (1.38 - 5.62)
Whole liver CTV (ml)	1493.5 (1106 - 2052)
BMI	23.74 (20.42 - 29.12)

Median graft versus recipient weight ratio was 1.12%, and ranged from 0.81 to 1.45%. Recipient´s estimated standard liver volume ranged from 948 to 1471g (median 1277g). The median relation between GW and estimated standard liver volume was 62.45 (44.23-80.57). 

**FIGURE 2:**
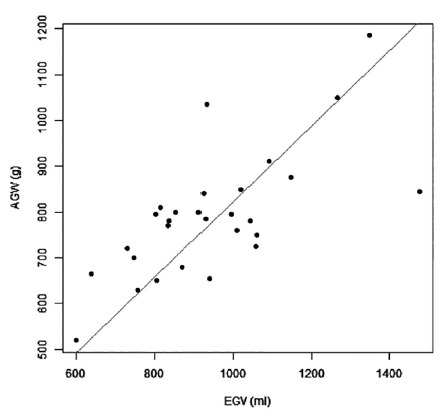
Positive linear correlation between estimated graft volume (EGV) and actual graft weight (AGW)

Median actual GW was 782.5 g, averaged 791.43±136 g, and ranged from 520 to 1185g. Median estimated GV was 927.5 ml, averaged 944.86±200.74 ml, and ranged from 600 to 1477 ml. Linear regression of estimated GV and actual GW were significantly linear (AGW=0.82EGV, r2=0.98, slope=0.47, standard deviation of 0.024 and p<0.0001) as illustrated in Figure 2. Spearman linear correlation was 0.65 with 95% CI of 0.45-0.99 (p<0.0001).

## DISCUSSION

An accurate preoperative GV estimation is essential in selecting suitable donors for adequate recipients of LDTL. It can provide valuable information to keep a safe remnant liver volume to the donor as well as reducing the recipient´s postoperative morbidity and mortality. 

LDTL recipients should receive sufficient hepatic parenchyma. Ideally relationship between weight of the graft and weight of the recipient should be about 0.8-1% to avoid small for size syndrome^1^ ^,^ ^10^. Several studies have shown that weight of the graft and weight of the recipient ratio below 0.8 are associated with higher initial postoperative mortality (3-months survival of 54%)^10^. Other factors, such as portal venous pressure over 15 mmHg^13^ and excessive portal vein inflow (over than 250 ml/min/100g of liver weight)^8^ are also associate with poor outcomes.

Therefore, predicting actual graft weight with pre-operative radiological evaluation is essential to define if LDLT is feasible. CTV is widely used as the standard method for preoperative estimation of hepatic graft weight^9^. However, it has been shown that CTV tends to overestimate both graft volume and weight^12^. Another concern is about health liver density; usually hepatic parenchyma volumetric measurements are compared based on the assumption that density is 1.00 g/ml. However, the relation between graft volume and GW is not exact and the one-to-one rule should not be applied for all patients. Yoneyama et al.^22^ have found correlation coefficients between estimated GV and GW in right lobe grafts of 0.84 and left lobe of 0.85^22^. They have emphasized that it could not be applicable in others institutions due to several biases. 

Several factors can interfere in graft weight analysis, such as: 1) the hepatic transection line defined by ischemic area, and/or anatomical parameters; and 2) the timing to graft weight, since high osmotic preservation solutions can induce cellular dehydration causing significant weight reduction in liver grafts^5^ ^,^ ^7^. Recently, Satou et al.^17^ have shown a reduction of up to 10% of the initial weight post-hepatectomy after back-table surgery has been completed^17^.

Accordingly, there are many strategies to estimate right graft weight and volume (Table 2). In our review, were found at least 11 different^2^ ^,^ ^3^ ^,^ ^4^ ^,^ ^6^ ^,^ ^7^ ^,^ ^8^ ^,^ ^9^ ^,^ ^11^ ^,^ ^12^ ^,^ ^17^ ^,^ ^18^ ^,^ ^21^ ^,^ ^22^, and sometimes conflicting, mathematical formulas aiming to predict graft size. Some of these methods do not use the computed tomography volumetry, but body surface area, and the diameter of the portal vein and/or their branches. This wide variation could be explained by donor´s heterogeneity due to age, gender, regional demographics, inclusion or not of the medium hepatic vein in the graft, different types of preservation solution, among others. The main purpose of our study was to determine the correlation between EGV and AGW in our center. We found that 1.00 ml of preoperative CTV correlates with 0.82 g of graft weight. Then, we believe that use the coefficient of 0.82 can predict more accurately the right hepatic graft weight in our population. 

**TABLE 2:** Studies evaluating right graft volume or weight estimation.

Author ^ref^	Year	Country	Formula	Patients enrolled	Type of graft	Solution
Hwang et al.^6^	2002	Korea	GW = EGV /1.22	12	Right lobe	HTK
Gondolesi et al.^4^	2004	USA	RGV= 440 X BSA	91	Right lobe	UW
Lemke et al.^12^	2006	Germany	RGW=(0.678 x EGV)+143.704	16	Right lobe	HTK
Khalaf et al.^8^	2007	Saudi Arabia	RGV= EGV x SLV	28 3	Right lobe Extended right lobe§	-
Kayashima et al.^7^	2008	Japan	GV = 70.767 + [0.703 x EGV] + (1.298 x donor age)	139 77	Left lobe Right lobe*	UW
Kim et al.^9^	2010	Korea	RGW = (0.8815 x EGV Blood Free ) + 88.5117	88	Right lobe	HTK
Chan et al.^2^ ^,^ ^3^	2011	China	RGW = EGV / 1.19	285	Extended right lobe§	HTK
Lee et al.^11^	2011	Taiwan	RGV=[R^2^/ (R^2^ +L^2^)] x100%	4	Full right (split)	HTK
Wang et al.^21^	2011	Taiwan	RGV = SLV x [R^2^/(R^2^ + L^2^)]	175	Right lobe	HTK
Yoneyama et al.^22^	2011	Japan	RGW = 0.84 x EGV	39	Right lobe	HTK/UW
Tongyoo et al.^18^	2012	USA	RGV = SLV × [(RA^2^+RP^2^) / (RA^2^+RP^2^+L^2^)]	200	Right lobe	HTK
Present study	2014	Brazil	RGW = 0.82 x EGV	28	Right lobe	HTK

* including right lobes, extended right lobe and 1 posterior segment.; § including medium hepatic vein. Abbreviation: BSA =body surface area, calculated by Mostellerxxxx formula: square root (weight [Kg] x height [cm]/3600); EGV=estimated graft volume by CT volumetry; GV=graft volume; GW=graft weight; L =left portal; vein diameter; R =right portal vein diameter; RA=right anterior portal vein diameter; RP=right posterior portal vein diameter; RGV=right graft volume ; RG=right graft weight; SLV=stand liver volume calculated by Makuuchixxx formula (706.2 x body surface area [m2] + 2.4).

## CONCLUSION

The one-to-one rule did not apply in patients with normal liver parenchyma. A better estimation of graft weight could be reached by multiplying estimated graft volume by 0.82.
